# Tests for Categorical Data Beyond Pearson: A Distance Covariance and Energy Distance Approach

**DOI:** 10.1002/bimj.70129

**Published:** 2026-06-08

**Authors:** Fernando Castro‐Prado, Wenceslao González‐Manteiga, Javier Costas, Fernando Facal, Dominic Edelmann

**Affiliations:** ^1^ Department of Statistics, Faculty of Mathematics University of Santiago de Compostela Santiago de Compostela Galicia Spain; ^2^ Psychiatric Genetics Laboratory, Santiago Health Research Institute (IDIS) University Hospital Santiago de Compostela Galicia Spain; ^3^ Biostatistics Department German Cancer Research Center (DKFZ) Heidelberg Baden‐Württemberg Germany

**Keywords:** categorical data, contingency tables, distance covariance, independence testing, Pearson's chi‐squared test

## Abstract

Categorical variables are of uttermost importance in biomedical research. When two of them are considered, it is often the case that one wants to test whether or not they are statistically dependent. We show weaknesses of classical methods—such as Pearson's and the G‐test—and we propose testing strategies based on distances that lack those drawbacks. We first develop this theory for classical two‐dimensional contingency tables, within the context of distance covariance, an association measure that characterizes general statistical independence of two variables. We then apply the same fundamental ideas to one‐dimensional tables, namely, to the testing for goodness of fit to a discrete distribution, for which we resort to an analogous statistic called energy distance. We prove that our methodology has desirable theoretical properties, and we show that we can calibrate the null distribution of our test statistics without resampling. We illustrate all this in simulations, as well as with some real data examples, demonstrating the adequate performance of our approach for biostatistical practice.

## 1 Introduction

In previous work by us (Castro‐Prado et al. 2026), an interesting data set from complex disease genomics motivated us to define distances on discrete spaces of cardinality 3 and test independence among variables whose support lies on such spaces. Since the times of Karl Pearson (more than a century ago), the corresponding test for categorical variables with an arbitrary finite number of categories has been of paramount interest to manifold applications. As a matter of fact, independence of categorical variables ranks among the most often tested hypotheses in biomedical practice (Berrett and Samworth 2021). Discrete data arise in health sciences in a variety of contexts (Agresti 2019; Preisser and Koch 1997)—for measuring responses to treatments, signposting the stage of a disease (or whether the disease is present), establishing subgroups after a diagnosis, and so forth.

In this paper, we present the distance and kernel counterpart (Edelmann and Goeman 2022) of what Pearson (1900) did. We derive some theory for independence testing and extend it to the problem of goodness of fit. We finally illustrate the performance of our methodology with synthetic and real data examples, including the comparison with competing methods.

For independence, we will consider categorical variables X∈{1,…,I} and Y∈{1,…,J}. Given an IID sample ${\left\{\left(X_{m},Y_{m}\right)\right\}}_{m=1}^{n}$, one can construct the I×J contingency table ${\left(n_{ij}\right)}_{i,j}$ by counting the observations per pair of categories (X,Y):

$$n_{ij}=\sum_{m=1}^{n}1_{\left\{X_{m}=i,Y_{m}=j\right\}}.$$

Under the null hypothesis, we expect to observe, in each cell

$$n_{ij}^{*}:=\frac{1}{n}\sum_{k=1}^{J}n_{ik}\sum_{k=1}^{I}n_{kj}.$$

One of the most common test statistics is Pearson's

$$\chi^{2}=\sum_{i=1}^{I}\sum_{j=1}^{J}\frac{{\left(n_{ij}-n_{ij}^{*}\right)}^{2}}{n_{ij}^{*}},$$

for which the p‐values are either computed using a chi‐squared distribution with (I−1)(J−1) degrees of freedom, or with permutations. The same holds for the null distribution of the G‐test:

$$G=2\sum_{i=1}^{I}\sum_{j=1}^{J}n_{ij}\log\left(\frac{n_{ij}}{n_{ij}^{*}}\right),$$

which is essentially the likelihood ratio test for this problem (Agresti 2019, Section 2.4.1). Other available methods include Fisher's exact test (Fisher 1934) and the U‐statistic permutation test (Berrett and Samworth 2021). The authors of this last work very illustratively show how classical methods have important limitations related to imbalanced cell counts, which justifies the need for new techniques for such a relevant problem.

For the problem of goodness of fit, it is customary to resort to Pearson's (chi‐squared) test, for which the philosophy is, once more, “the squared difference of the observed and the expected, divided by the expected;” now with the difference that the table is 1×I and the expected cell counts will be

$$n_{i}^{*}=nP_{H_{0}}\left\{X=i\right\}\;.$$

The scope of this work will be to address the testing for independence and goodness of fit with categorical data, using the aforementioned techniques, collectively known as *energy statistics* (Székely and Rizzo 2017). The remainder of the paper is organized as follows. Section 2 contains our novel approach to the testing for independence between two categorical variables. In Section 3, we develop the testing for goodness of fit to a discrete distribution using the same basic notions, but with different theoretical tools. Some illustrative simulations are reported in Section 4. In Section 5, we apply the method to real data, to show applicability. Concluding remarks are given in Section 6. Proofs for our theoretical results are given in Appendices A and B.

## 2 The Distance Covariance Test of Independence Between Two Categorical Variables

Given an IID sample ${\left\{\left(X_{m},Y_{m}\right)\right\}}_{m=1}^{n}$ of (X,Y), a consistent (but biased) estimator for the generalized distance covariance (Székely and Rizzo 2017) between our two jointly distributed random variables is given by

$$\hat{V}={\hat{T}}_{1}-2{\hat{T}}_{2}+{\hat{T}}_{3},$$

where

$$\begin{array}{cc} {\hat{T}}_{1} & =\frac{1}{n^{2}}\sum_{l,m=1}^{n}d_{X}\left(X_{l},X_{m}\right)\;d_{Y}\left(Y_{l},Y_{m}\right),\\ {\hat{T}}_{2} & =\frac{1}{n^{3}}\sum_{l=1}^{n}\left(\sum_{m=1}^{n}d_{X}\left(X_{l},X_{m}\right)\right)\;\left(\sum_{m=1}^{n}d_{Y}\left(Y_{l},Y_{m}\right)\right),\\ {\hat{T}}_{3} & =\frac{1}{n^{4}}\left(\sum_{l,m=1}^{n}d_{X}\left(X_{l},X_{m}\right)\right)\;\left(\sum_{l,m=1}^{n}d_{Y}\left(Y_{l},Y_{m}\right)\right). \end{array}$$

We assume that the supports X and Y of X and Y, respectively, are finite, with cardinality $I\in Z^{+}$ and $J\in Z^{+}$. When it comes to deciding which (pseudo)metrics $d_{X}$ and $d_{Y}$ to equip them with, the only restriction we have for distance covariance and associated techniques to work out is that we need to be in a (pseudo)metric structure of strong negative type (Castro‐Prado and González‐Manteiga 2020). Now the question would be which of those feasible distances is the most convenient to use. Since we are working with categorical data and we want to be as agnostic as possible in terms of the underlying relationships among categories, in the following we will restrict ourselves to the case in which the metric structure on both marginal spaces reflects this agnosticism. In other words, we will equip both X and Y with the discrete distance (which we will henceforward denote simply as d for both spaces):

$$d\left(z,z^{\prime }\right)=1-\delta_{z\;z^{\prime }}=1_{\left\{z\neq z^{\prime }\right\}},$$

where $\delta_{\cdot \;\cdot }$ denotes the Kronecker delta and $\left(z,z^{\prime }\right)$ is either in X×X or in Y×Y. Alternatively, we could obtain the same test statistic by identifying the I categories of X with an orthonormal basis of $R^{I}$ and then using the Euclidean distance and classical distance covariance (Székely et al. 2007), instead of its extension to metric spaces (Jakobsen 2017; Lyons 2013).

We now construct the I×J contingency table for the sample ${\left\{\left(X_{m},Y_{m}\right)\right\}}_{m=1}^{n}$ of (X,Y). Its (i,j)th cell will be denoted by $n_{ij}$:

$$n_{ij}=\sum_{m=1}^{n}1_{\left\{X_{m}=i,Y_{m}=j\right\}}.$$

We call the $n_{ij}$’s *observed* cell counts, whereas their *expected* counterparts are their expected values under the null hypothesis (i.e., independence of X,Y).

We now introduce the notation $n_{i\cdot }$ and $n_{\cdot j}$ for the row and column sums of the contingency table

$$n_{i\cdot }:=\sum_{j=1}^{J}n_{ij}=\sum_{m=1}^{n}1_{\left\{X_{m}=i\right\}};$$

$$n_{\cdot j}:=\sum_{i=1}^{I}n_{ij}=\sum_{m=1}^{n}1_{\left\{Y_{m}=j\right\}}.$$

These allow us to define the expected cell counts (under independence):

$$n_{ij}^{*}=\frac{1}{n}n_{i\cdot }n_{\cdot j}.$$

By performing some algebraic manipulations, one can see that our test statistic can compactly be written as

(1) $$\hat{V}=\frac{1}{n^{2}}\sum_{i=1}^{I}\sum_{j=1}^{J}{\left(n_{ij}-n_{ij}^{*}\right)}^{2}.$$

On the other hand, Pearson's (chi‐squared) test for independence is based on the statistic

$$\chi^{2}=\sum_{i=1}^{I}\sum_{j=1}^{J}\frac{{\left(n_{ij}-n_{ij}^{*}\right)}^{2}}{n_{ij}^{*}},$$

which only differs in a “normalizing” denominator in each term of the sum.

We now state the following result on the null distribution of our test statistic (1). A self‐contained proof can be found in Appendix A, but we would like to point out that general theory for degenerate V‐statistics (de Wet 1987) also allows to derive an asymptotic null distribution for the same statistic.

Theorem 2.1 Let $\left(X_{1},\text{…},X_{n}\right)$ and $\left(Y_{1},\text{…},Y_{n}\right)$ be IID samples of jointly distributed random variables (X,Y)∈{1,2,…,I}×{1,2,…,J}, with $q_{i}:=P\left(X=i\right)$ and $r_{j}:=P\left(Y=j\right)$.

Consider X and Y equipped with the discrete metric. Then, the empirical distance covariance between the two random variables can be written as

$${\hat{\mathrm{dCov}}}_{\text{discrete}}^{2}\left(X,Y\right)=\frac{1}{n^{2}}\sum_{i=1}^{I}\sum_{j=1}^{J}{\left(n_{ij}-n_{ij}^{*}\right)}^{2}.$$

In addition, whenever X and Y are independent, for n→∞,

$$n\;{\hat{\mathrm{dCov}}}_{\text{discrete}}^{2}\left(X,Y\right)\overset{D}{⟶}\sum_{i=1}^{I-1}\sum_{j=1}^{J-1}\lambda_{i}\mu_{j}Z_{ij}^{2},$$

where $Z_{ij}^{2}$ are independent chi‐squared variables with one degree of freedom each. $\lambda_{1},\text{…},\lambda_{I}$ are the eigenvalues of matrix $A={\left(a_{ij}\right)}_{I\times I}$, whose entries are

$$a_{ij}=q_{i}\delta_{ij}-q_{i}q_{j},$$

where $\delta_{ij}$ is the Kronecker delta. Similarly, $\left\{\mu_{1},\text{…},\mu_{J}\right\}$ is the spectrum of $B={\left(b_{ij}\right)}_{J\times J}$, with

$$b_{ij}=r_{i}\delta_{ij}-r_{i}r_{j}.$$

It should be noted that A and B are the covariance matrices of a multinomial distribution multiplied by a factor (actually, of a “multi‐Bernoulli” distribution).

In practice, when it comes to using the distribution above, we will take the empirical estimators ${\hat{q}}_{i}$ and ${\hat{r}}_{j}$, then construct estimators of A and B from them, to finally use the products of their eigenvalues as the coefficients in the linear combination of IID $\chi_{1}^{2}$’s.

Hence, obtaining the p‐values of our test boils down to evaluating the distribution function of weighted sums of chi‐squared variables. The approximation of quadratic forms of Gaussian variables has been very well studied historically and it arises fairly often in statistical practice (Duchesne and Lafaye de Micheaux 2010). The algorithm by Imhof (1961) is arguably one of the best known ones, but its speed can come at the price of precision (Goeman et al. 2011). We have instead chosen to resort to Farebrother (1984) for our approximations, in the implementation by Duchesne and Lafaye de Micheaux (2010).

## 3 The Energy Test for Goodness of Fit to a Discrete Distribution

Let us once again consider a categorical variable X with support X of cardinality $I\in Z^{+}$, which we will assume to be {1,…,I} without loss of generality. We observe a sample $X_{1},\text{…},X_{n}$ IID X and we will use it to test for X∼F having been drawn from a certain distribution $F_{0}$:

$$H_{0}:F=F_{0}.$$

The distance‐based statistic for this kind of test would be the adaptation of the one by Székely and Rizzo (2005) to our setting. Let d denote once more the discrete distance on the support of X. Then, the energy distance between the empirical distribution and F (which equals $F_{0}$ under the null hypothesis) is

$$E_{n}=n\left[\frac{2}{n}\sum_{l=1}^{n}Ed\left(x_{l},X\right)-Ed\left(X,X^{\prime }\right)-\frac{1}{n^{2}}\sum_{l,m=1}^{n}d\left(x_{l},x_{m}\right)\right];$$

where ${\left\{x_{l}\right\}}_{l=1}^{n}$ is a sample realization of ${\left\{X_{l}\right\}}_{l=1}^{n}$ and $X^{\prime }$ is an IID copy of X. We refer the reader to Rizzo and Székely (2016) for a more comprehensive review on this kind of statistics.

If we now define $p_{i}:=P_{H_{0}}\left\{X=i\right\}$ (for i=1,…,I), we have that the expected cell count for each category is $n_{i}^{*}:=np_{i}$, whereas the observed cell count is simply

$$n_{i}:=\sum_{l=1}^{n}1_{\left\{X_{l}=i\right\}}.$$

With this notation, and after some algebra, we can write our test statistic for $H_{0}:F=F_{0}$ as

$$E_{n}=\frac{1}{n}\sum_{i=1}^{I}{\left(n_{i}-n_{i}^{*}\right)}^{2},$$

which again resembles Pearson's without its denominator. As of its null distribution, we present the following result.

Theorem 3.1 Let $\left(X_{1},\text{…},X_{n}\right)$ be an IID sample of random variable X∈X={1,2,…,I}.

Consider X equipped with the discrete metric. Then, the energy distance test statistic for goodness of fit to a fixed distribution $p={\left(p_{i}\right)}_{i=1}^{I}$ on {1,…,I} is

$$E_{n}=\frac{1}{n}\sum_{i=1}^{I}{\left(n_{i}-n_{i}^{*}\right)}^{2},$$

with the observed counts being $n_{i}:=\sum_{l=1}^{n}1_{\left\{X_{l}=i\right\}}$ and the expected ones: $n_{i}^{*}=np_{i}$.

Then, whenever X is distributed according to p, for n→∞,

$$E_{n}\overset{D}{⟶}\sum_{i=1}^{I-1}\lambda_{i}Z_{i}^{2},$$

where $Z_{i}^{2}$ are independent chi‐squared variables with one degree of freedom each. $\lambda_{1},\text{…},\lambda_{I}$ are the eigenvalues of matrix $C={\left(c_{ij}\right)}_{I\times I}$ with

$$c_{ij}=p_{i}\delta_{ij}-p_{i}p_{j},$$

where $\delta_{ij}$ is the Kronecker delta.

Note that, matrix C here is, once again, a covariance matrix of a multinomial, and therefore has zero as one of its eigenvalues and I−1 as its rank.

For the proof of the preceding theorem, we forward the reader to Appendix B.

## 4 Simulation Study

We will now show how the tests proposed in Sections 2 and 3 perform numerically, by simulating some population models that we consider illustrative. Subsection 4.1 is devoted to the distance covariance test and Subsection 4.2, to the one based on the energy distance.

### 4.1 Distance Covariance Test of Independence

As previously mentioned, the test statistic we present in Section 2 is (almost) the same as the USP test statistic by Berrett and Samworth (2021), with the substantial—albeit not fundamental—difference being that theirs is the U‐statistic counterpart of our V‐statistic. The approach for the testing, however, is completely different, since they use permutations, whereas we derive the (asymptotic) null distribution of the test statistic (Theorem 2.1). Nevertheless, applying classical U‐statistic theory (Lee 1990, Section 3.2.2), one can see that the USP test statistic (times the sample size) has as its asymptotic null distribution the same quadratic form described in Theorem 2.1 for distance covariance.

We will therefore use the family of models for contingency tables with exponentially decaying marginals described by Berrett and Samworth (2021), as it provides a good framework for assessing both the calibration of significance and the performance in terms of power. We will compare our method with theirs, as well as with the asymptotic distribution of the USP, Pearson's chi‐squared test, Pearson's test with permutations, Fisher's exact test, and the G‐test.

Let us first define the model. For fixed I and J, we define the cell probabilities of our contingency table under independence as

$$p_{ij}^{\left(0\right)}:=\frac{2^{-(i+j)}}{\left(1-2^{-I}\right)\left(1-2^{-J}\right)}\text{; for}\;i=1,\text{…},I;j=1,\text{…},J.$$

The above expression is clearly the product of the marginal probabilities. It is also easy to see that the probability mass is maximized in the top‐left corner of the contingency table and it decreases rightward and downward.

Now, for each $\varepsilon \in R^{+}$ small enough so that no probabilities are out of [0,1], we define $p_{ij}^{\left(\varepsilon \right)}$ as the following perturbation of $p_{ij}^{\left(0\right)}$:

$$p_{ij}^{\left(\varepsilon \right)}:=\left\{\begin{array}{cc} p_{ij}^{\left(0\right)}+\varepsilon & \text{if}\;(i,j)\in \{(1,1),(2,2)\}\\ p_{ij}^{\left(0\right)}-\varepsilon & \text{if}\;(i,j)\in \{(1,2),(2,1)\}\\ p_{ij}^{\left(0\right)} & \text{otherwise}\;, \end{array}\right.$$

where $\varepsilon \leq \min\;\left\{\;{\left[8\left(1\;-2^{-I}\right)\left(1\;-2^{-J}\right)\right]}^{-1}\;,1\;-\;{\left[4\left(1\;-\;2^{-I}\right)\left(1\;-\;2^{-J}\right)\right]}^{-1}\right\}$. The larger ε is (within its range), the further the contingency table is from the null hypothesis. The upper bound for ε can be arbitrarily close to 0.125 (as both I and J tend to infinity), but for us it will be approximately $\frac{1024}{7905}\approx 0.1295$, as we will be restricting our simulated contingency tables to the dimensions we state below.

To follow exactly the footprints of Berrett and Samworth (2021), we consider $M=10^{4}$ replicates of contingency tables with I=5 rows and J=8 columns, containing n=100 observations. For each of the methods based on permutations, we chose B=999 as the number of resamples and we use the algorithm by Patefield (1981) to uniformly draw the contingency tables with given marginals.

**FIGURE 1:**
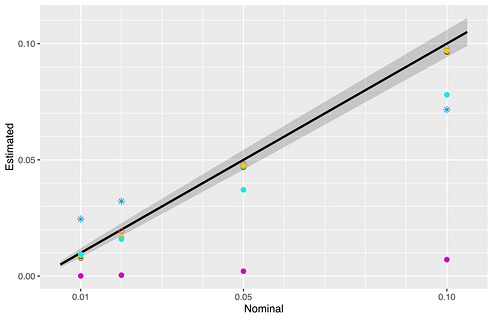
Empirical power under the null hypothesis ($\hat{\alpha }$) versus nominal significance level (α), for the decaying marginals model, comparing our distance covariance method (golden points), Pearson's chi‐squared test (pale blue), Pearson's test with permutations (dark red), the USP (black), the USP with the asymptotic approximation (turquoise), Fisher's exact test (green), and the G‐test (purple). The gray shadow is a 95 % confidence band for $\hat{\alpha }$ given α.

For ε=0, we can see how we calibrate significance. Figure 1 shows the results with our method for some reference values of nominal α, and allows for a comparison with competing techniques. We see that we control type I error very satisfactorily, both when considering our results only and when comparing them with Pearson's test with permutations, the USP and Fisher's exact test. All the aforementioned tests perform satisfactorily in terms of calibration of α. The G‐test, however, proves to be far too conservative. Pearson's chi‐squared fails, too, when it comes to controlling the type I error, but does so in a less dramatic fashion (and it actually produces a good result for nominal α of 0.05). To find an explanation to this phenomenon, one should note that the model we are using features very small expected cell counts, which will tend to break down the heuristic rules as to when to use the chi‐squared distribution with (I−1)(J−1) degrees of freedom to compute p‐values or not.

**FIGURE 2:**
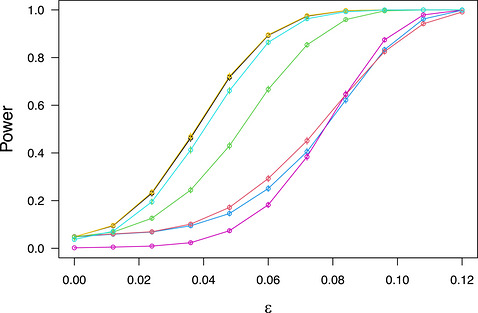
Power curve comparison for the decaying marginals model, displaying our distance covariance method (golden curve), Pearson's chi‐squared test (pale blue), Pearson's test with permutations (dark red), the USP (black), the USP with asymptotic approximation (turquoise), Fisher's exact test (green), and the G‐test (purple). The 5×8 cells of each contingency table were filled with n=100 observations. $M=10^{4}$ replicates were considered. Error bars span from −3 to +3 standard deviations for each value of parameter ε, which indicates the distance from the null hypothesis.

In terms of power, Figure 2 shows that we perform very similarly to the USP (which shows how our derivation of the null distribution is correct and that the asymptotic approximation is not very far off when n=100). The power curve of Fisher's exact test is clearly under ours, whereas the one for the remaining classical methods is quite low for most values of ε.

Other than the theoretical insight that using distance covariance provides (i.e., characterizing general independence, the relationship to kernels and global tests, and so forth), we provide a relevant practical improvement with respect to the USP—running time. Our experiments show that we are 3 orders of magnitude faster in testing than the USP. This remarkable difference in speed is not due to anything being intrinsically slow about computing the USP statistic, but it is simply a consequence of comparing a testing approach that uses a closed‐form null distribution with another one that requires almost a thousand permutations in its default settings (Berrett and Samworth 2021). All this refers to the USP as provided by its original authors. When resorting to its asymptotic null distribution, as the one we are presenting for the distance covariance, no relevant discrepancy in computation times between the two methods is observed.

### 4.2 Energy‐Distance Test of Goodness of Fit

We will first summarize the notion of Hardy–Weinberg equilibrium (HWE), an important genetic concept that was independently introduced in 1908 by the eponymous authors (Hardy 1908; Weinberg 1908). Let us consider a biallelic locus, whose alleles we will denote as $A_{1}$ and $A_{2}$. Under panmixia and in the absence of evolutionary influences, the frequencies of both alleles and of each possible genotype ($A_{1}A_{1}$, $A_{1}A_{2}$, and $A_{2}A_{2}$) remain constant from generation to generation. If we use the following notation for the allele frequencies:

$$\theta_{1}:=f\left(A_{1}\right);\;\;\;\theta_{2}:=f\left(A_{2}\right);$$

the genotype frequencies that are to be maintained under the HWE are

$$f\left(A_{1}A_{1}\right)=\theta_{1}^{2};\;\;\;f\left(A_{1}A_{2}\right)=2\theta_{1}\theta_{2};\;\;\;f\left(A_{2}A_{2}\right)=\theta_{2}^{2};$$

where $\theta_{1}+\theta_{2}=1$. We point out that the *frequencies* that geneticists denote by f are what a statistician would call *proportions* in the population. It is also noteworthy that those frequencies that the HWE predicts are the terms of the expansion of

$${\left(\theta_{1}+\theta_{2}\right)}^{2}$$

as a sum.

**FIGURE 3:**
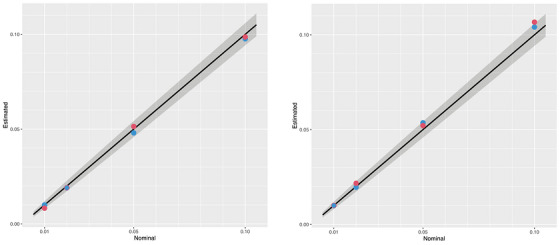
Empirical power under the null hypothesis ($\hat{\alpha }$) versus nominal significance level (α), for the goodness‐of‐fit test of the biallelic Hardy–Weinberg equilibrium, when $\theta_{1}=\frac{2}{3}$ (left‐hand plot) and $\theta_{1}=\frac{1}{2}$ (right). Red dots correspond to our energy distance method; blue are those for Pearson's chi‐squared test. The gray shadow is a 95 % confidence band for $\hat{\alpha }$ given α.

We will now start the simulations by showing the calibration of significance for some reference values of nominal α for our energy‐distance test and the chi‐squared test of goodness of fit. Based on the values for the allele frequencies we have encountered in the real data examples that we will be presenting in Subsection 5.2, we have chosen $\frac{2}{3}$ and $\frac{1}{2}$ as representative values of $\theta_{1}$ for our simulations. Figure 3 shows that both our method and the $\chi^{2}$ test perform well in terms of type I error. Every simulation in this subsection will take n=500 observations for each of the $M=10^{4}$ replicates. The sample size is a rounding of the one we have in Section 5, but our numerical experiments show qualitatively similar conclusions for other values of n.

We now introduce two models that depart from the null hypothesis. For model 2S, we first consider the HWE genotype frequencies for the case where $\theta_{1}=\frac{2}{3}$:

A1A1A1A2A2A2∫∫∫∫∫494919.

And we introduce a parameter s∈[0,1] which is zero under the null hypothesis and it increases as so does the distance to $H_{0}$:

A1A1A1A2A2A2∫∫∫∫∫4(1−s)94(1−s)91+8s9.

On the other hand, model 2K introduces parameter k∈[0,1], which increases as so does the divergence from HWE with $\theta_{1}=\theta_{2}=\frac{1}{2}$:

A1A1A1A2A2A2∫∫∫∫∫1−k4k+121−k4.

**FIGURE 4:**
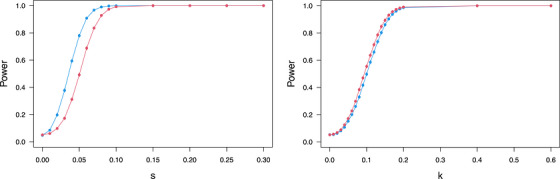
Power curve comparison for models 2S (left) and 2K (right), displaying our energy distance method (red lines and dots) and Pearson's chi‐squared test (blue). $M=10^{4}$ replicates with sample size n=500 were considered. Error bars are barely visible in this case, but they span from −3 to +3 standard deviations for each value of parameters s and k, which in turn indicate the distance to the null hypothesis.

We present power curves for models 2S and 2K for both E and the $\chi^{2}$ test in Figure 4. We observe that both tests perform very satisfactorily, even for divergences from the null hypothesis that are not the highest in magnitude.

In order not to restrict ourselves to the case where the number of categories is only 3, we will now generalize the notion of HWE. One way of doing so would be to increase the ploidy, which would yield as genotype frequencies the terms of the binomial expansion of

$${\left(\theta_{1}+\theta_{2}\right)}^{c}$$

for c>2. We will however opt for a generalization that one can encounter in humans, that is, increasing the number of possible alleles. Let us consider a triallelic locus with allele frequencies

$$\theta_{1}:=f\left(A_{1}\right);\;\;\;\theta_{2}:=f\left(A_{2}\right);\;\;\;\theta_{3}:=f\left(A_{3}\right);$$

where $\theta_{1}+\theta_{2}+\theta_{3}=1$. Then, the Hardy–Weinberg genotype frequencies are

A1A1A2A2A3A3A1A2A1A3A2A3∫∫∫∫∫θ12θ22θ322θ1θ22θ1θ32θ2θ3.

As with the biallelic case, we first consider a scenario where the allele frequencies are unbalanced: $\theta_{1}=0.70$, $\theta_{2}=0.25$, and $\theta_{3}=0.05$. Model 3S departs from the HWE for those values as parameter s∈[0,1] increases within its range:

A1A1A2A2A3A3A1A2A1A3A2A3∫∫∫∫∫0.49(1−s)1+15s160.0025(1−s)0.35(1−s)0.07(1−s)0.025(1−s).

And we also consider the case where $\theta_{1}=\theta_{2}=\theta_{3}=\frac{1}{3}$. By introducing parameter k∈[0,1] to tune the intensity of the departure from the null, we define model 3K:

A1A1A2A2A3A3A1A2A1A3A2A3∫∫∫∫∫2k+192k+192k+192−2k92−2k92−2k9.

**FIGURE 5:**
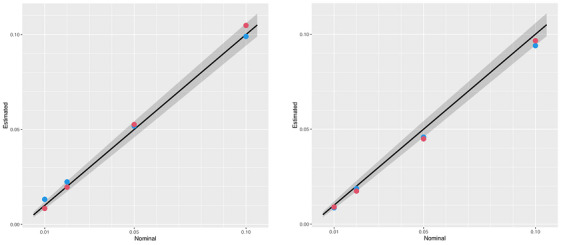
Empirical power under the null hypothesis ($\hat{\alpha }$) versus nominal significance level (α), for the goodness‐of‐fit test of the triallelic Hardy–Weinberg equilibrium, when $\left(\theta_{1},\theta_{2},\theta_{3}\right)=\left(0.70,0.25,0.05\right)$ (left‐hand plot) and $\theta_{1}=\theta_{2}=\theta_{3}=\frac{1}{3}$ (right). Red dots correspond to our energy distance method; blue are those for Pearson's chi‐squared test. The gray shadow is a 95% confidence band for $\hat{\alpha }$ given α.

**FIGURE 6:**
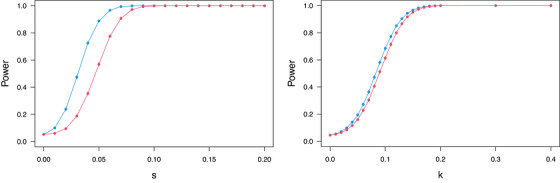
Power curve comparison for models 3S (left) and 3K (right), displaying our energy distance method (red lines and dots) and Pearson's chi‐squared test (blue). $M=10^{4}$ replicates with sample size n=500 were considered. Error bars are barely visible in this case, but they span from −3 to +3 standard deviations for each value of parameters s and k, which in turn indicate the distance from the null hypothesis.

Figure 5 shows that, once again, both the energy distance and Pearson's chi‐squared control type I error. The power curves in Figure 6 show E a bit below the $\chi^{2}$, but we do not perform a great deal worse.

## 5 Real Data Analyses

To complete the numerical analyses in Section 4, we now demonstrate the applicability of the methodology introduced in this paper. We introduce two examples of interest to biomedical practice that arise from a data set produced by us (Facal et al. 2022). Subsection 5.1 explores the potential of our distance covariance independence test for interpreting the clinical significance of polygenic scores, whereas Subsection 5.2 presents real‐life examples of the Hardy–Weinberg models introduced in Subsection 4.2.

### 5.1 Distance–Covariance Test of Independence

We begin by showing with a real biomedical example how our test for dependence can be used in practice. We consider data from Facal et al. (2022), where we observe n=427 patients of schizophrenia. For each of them, we consider a categorical variable X indicating how chronic the psychiatric disorder is in that person (an index with four possible values, based on the admission history in health facilities), and another categorical variable Y which indicates the PRS tercile (i.e., whether the *polygenic risk score* for schizophrenia of the patient is low, medium, or high).

Although the clinical utility of PRSs is very limited at the individual level, they may be useful for the identification of specific quantiles of risk for stratification of a population to apply specific interventions (Torkamani et al. 2018). This is why it makes the most sense to consider PRS as a categorical variable (and not one with many categories) instead of working with its raw individual scores. The data for our example can be seen in Table 1.

**TABLE 1:** Contingency table for the chronicity data set.

Chr. \ PRS	$T_{1}$	$T_{2}$	$T_{3}$	
Low	12	9	4	25
Middle‐low	37	20	29	86
Middle‐high	40	58	44	142
High	53	55	66	174
	142	142	143	427

We can now apply the different methods of Section 4 to our data set. Pearson's test yields similar results with and without permutations, due to the lack of low (expected) cell counts. In both cases, the p‐value is around 0.025 and one would reject independence for a nominal α of 0.05. The G‐test offers a p of 0.022, in line with Pearson's. Fisher's exact test also does not diverge much, with 0.024. Finally, the USP and distance covariance yield p‐values of 0.046 and 0.045. All things considered, in this case one would tend to reject the null hypothesis of independence (when α=0.05), which is consistent with the hypothesis that the PRS can measure how “sick” a patient is (or, more generally, how intense the trait of interest is).

### 5.2 Energy‐Distance Test of Goodness of Fit

We will now see two examples of how one can test for goodness of fit with our methodology. Let us consider again the cohort of n=427 individuals by Facal et al. (2022). As previously mentioned, a frequent quality control for GWAS data is whether or not each SNP is in HWE in the control group.

We will first focus the biallelic SNP rs9545047 because it is one of the variants in the most current list of loci known to influence gene expression in relationship with schizophrenia, as per Extended Data Table 1 in Trubetskoy et al. (2022). This SNP has also the peculiarity of not being in a protein‐coding gene, but in one that is transcribed into long intergenic nonprotein coding RNA (lincRNA). For this locus, we observe genotype *AA* 139 times; *CA*, 232 times and *CC*, 56 times. Using the online tool UCSC Genome Browser (Nassar et al. 2023), we can retrieve some useful information about this SNP, including the allele frequencies according to the GnomAD database, which gives us

f(C)≈0.41.

GnomAD v4.1.0 offers allele frequencies for different ancestries, and we have chosen the value for European (non‐Finnish) population, since it is the best match for the geographical origin of our 427 individuals, which are from the northwestern Iberian peninsula. We have opted for GnomAD because it is the online resource for human population genetics with the largest sample size that we are aware of.

Therefore, the expected cell counts are

AACACC∫∫∫∫∫148.6206.671.8.

When applying our energy testing procedure, it yields a p‐value of 0.027, which coincides with the one obtained with Pearson's. This means that both tests would reject the null hypothesis for conventional nominal values of α like 0.05. This is a perfectly logical result for an SNP linked to schizophrenia, which is expected to have one of its haplotypes at a frequency that departs from the one that would be encountered under the HWE. One should also note that SNPs like this one are not left out during the quality control phase of the GWAS because the Hardy–Weinberg filter only applies to the control group (in our case, a pool of individuals not presenting schizophrenia).

Given that there are not many triallelic SNPs, we will just be considering one of them for illustrative purposes, without giving much profound interpretation to the results. We choose SNP rs2594292, for which the observed genotypes are

AAGGTTAGATTG∫∫∫∫∫2143401481615.

Once again resorting to GnomAD, we get the following population allele frequencies:

f(A)≈0.69;f(G)≈0.26;f(T)≈0.05.

Using them to calculate the expected cell counts, we get a p‐value of 0.24 with our method and of 0.07 with Pearson's. In this case, we observe more dissimilar results, but with none of the tests finding significant evidence of divergence from the HWE with nominal α of 0.05, which is a logical result for any SNP not known to be linked to schizophrenia.

## 6 Discussion and Conclusion

We have proposed a new test for the independence of categorical variables (one of the most often tested hypotheses in biomedical research) by using distance covariance, an association measure that characterizes general statistical independence. As we allow for arbitrary dimensions of the contingency table, this extends the possibilities we showed on previous work (Castro‐Prado et al. 2026) for the 3×3 case. We have also developed a novel testing strategy for the goodness of fit to a discrete distribution. For both methods, we demonstrate good performance and applicability, with simulations and analyses of relevant biomedical examples.

The test statistic we derive for independence happens to have a simple algebraic expression similar in spirit to that of Pearson's $\chi^{2}$ test. We are not the first to see the connection between the two tests, as it was already mentioned in Remark 3.12 of Lyons (2013) and explored in some detail in the final section of Edelmann and Goeman (2022). Nevertheless, the proofs we provide are original and we are the first ones (to our knowledge) to analyze the matter in detail. On top of that, we are not aware of any previous instance in the literature where a test for goodness of fit to a discrete distribution is built based on energy statistics.

Another test for independence that is related to ours is the one in Berrett and Samworth (2021), initially introduced in Berrett et al. (2021). The main conceptual difference in our approaches is that we derive the asymptotic null distribution of our V‐statistic and are able to satisfactorily use it in practice, whereas their testing is based on permutations (of a U‐statistic). It is also noteworthy that, in that article, no mention is made of distance‐based association measures, a relationship that we thoroughly explore. In return, we obtain from their results the conclusion that our test statistic is very close to being the minimum‐variance unbiased estimator of the population USP‐divergence statistic. As they indicate, if one assumes that the population quantity is meaningful (and we now know it is, given its connection to distance covariance), then the test statistic is a very good estimator of it.

A remarkable pragmatical difference between our goodness‐of‐fit test and the one for independence is that the former does not require to plug in any frequencies to then estimate the multinomial covariance matrix and get the coefficients of the linear combination of chi‐squared's. In this case, the $p_{i}$’s are fixed and known, since they are given by the null hypothesis. However, when testing whether or not the population distribution belongs to a certain family of distributions, one would need to plug in the parameters in which the family is indexed. The effect that the estimation of such parameters has in U‐ and V‐statistics has been studied by authors such as de Wet and Randles (1987) and Jimémez‐Gamero et al. (2003).

All in all, we have presented new methodology to address important problems of practitioners, proven solid theoretical properties, explored connections with well‐known methods, and illustrated all of it in simulated and real data sets. Future and current lines of work include extending these techniques to the study of associations between categorical and continuous data (Edelmann et al. 2024).

## Conflicts of Interest

The authors declare no conflicts of interest.

## Open Research Badges

This article has earned an Open Data badge for making publicly available the digitally‐shareable data necessary to reproduce the reported results. The data is available in the Supporting Information section.

This article has earned an open data badge “**Reproducible Research**” for making publicly available the code necessary to reproduce the reported results. “The results reported in this article could fully be reproduced.”

## Supporting information


**Supporting File:** bimj70129‐sup‐0001‐DataCode.zip.

## Data Availability

The data that are analyzed are contained in the text of the article already, since they comprise only a few observations of discrete variables.

## Appendix A Proof of Theorem 2.1

We will first show that the distance covariance test statistic has the compact form similar to Pearson's that we stated in the main manuscript, to then prove the asymptotic null distribution.

We will investigate the terms ${\hat{T}}_{1},{\hat{T}}_{2},{\hat{T}}_{3}$ one by one, to then see how $\hat{V}$ can be written as a simple expression.

$$\begin{array}{cc} {\hat{T}}_{1} & =\frac{1}{n^{2}}\sum_{l,m=1}^{n}d\left(X_{l},X_{m}\right)\;d\left(Y_{l},Y_{m}\right)\\ & =\frac{1}{n^{2}}\sum_{l,m=1}^{n}1_{\left\{X_{l}\neq X_{m},Y_{l}\neq Y_{m}\right\}}\\ & =\frac{1}{n^{2}}\sum_{l,m=1}^{n}\left(1-1_{\left\{X_{l}=X_{m}\right\}}-1_{\left\{Y_{l}=Y_{m}\right\}}+1_{\left\{X_{l}=X_{m},Y_{l}=Y_{m}\right\}}\right)\\ & =1-\frac{1}{n^{2}}\sum_{i=1}^{I}n_{i\cdot }^{2}-\frac{1}{n^{2}}\sum_{j=1}^{J}n_{\cdot j}^{2}+\frac{1}{n^{2}}\sum_{i=1}^{I}\sum_{j=1}^{J}n_{ij}^{2}. \end{array}$$

For ${\hat{T}}_{2}$, we first observe that

$$\sum_{m=1}^{n}d\left(X_{l},X_{m}\right)=\sum_{m=1}^{n}\left(1-1_{\left\{X_{l}=X_{m}\right\}}\right)=n-n_{X_{l}\cdot }$$

and hence

$$\begin{array}{cc} {\hat{T}}_{2} & =\frac{1}{n^{3}}\sum_{l=1}^{n}\left(n-n_{X_{l}\cdot }\right)\left(n-n_{\cdot Y_{l}}\right)\\ & =\frac{1}{n^{3}}\sum_{i=1}^{I}\sum_{j=1}^{J}\left(n-n_{i\cdot }\right)\left(n-n_{\cdot j}\right)n_{ij}\\ & =1-\frac{1}{n^{2}}\sum_{i=1}^{I}n_{i\cdot }^{2}-\frac{1}{n^{2}}\sum_{j=1}^{J}n_{\cdot j}^{2}+\frac{1}{n^{3}}\sum_{i=1}^{I}\sum_{j=1}^{J}n_{i\cdot }n_{\cdot j}n_{ij}. \end{array}$$

Finally,

$$\sum_{l,m=1}^{n}d\left(X_{l},X_{m}\right)=\sum_{l=1}^{n}\left(n-n_{X_{l}\cdot }\right)=n^{2}-\sum_{i=1}^{I}n_{i\cdot }^{2}$$

and hence

$$\begin{array}{cc} {\hat{T}}_{3} & =\frac{1}{n^{4}}\left(n^{2}-\sum_{i=1}^{I}n_{i\cdot }^{2}\right)\left(n^{2}-\sum_{j=1}^{J}n_{\cdot j}^{2}\right)\\ & =1-\frac{1}{n^{2}}\sum_{i=1}^{I}n_{i\cdot }^{2}-\frac{1}{n^{2}}\sum_{j=1}^{J}n_{\cdot j}^{2}+\frac{1}{n^{4}}\sum_{i=1}^{I}\sum_{j=1}^{J}n_{i\cdot }^{2}n_{\cdot j}^{2}. \end{array}$$

When adding up the terms to obtain $\hat{V}$, the terms 1, $\frac{1}{n^{2}}\sum_{i=1}^{I}n_{i\cdot }^{2}$, and $\frac{1}{n^{2}}\sum_{j=1}^{J}n_{\cdot j}^{2}$ all cancel out and we obtain

$$\begin{array}{cc} \hat{V} & =\frac{1}{n^{2}}\sum_{i=1}^{I}\sum_{j=1}^{J}n_{ij}^{2}-\frac{2}{n^{3}}\sum_{i=1}^{I}\sum_{j=1}^{J}n_{i\cdot }n_{\cdot j}n_{ij}+\frac{1}{n^{4}}\sum_{i=1}^{I}\sum_{j=1}^{J}n_{i\cdot }^{2}n_{\cdot j}^{2}\\ & =\frac{1}{n^{2}}\sum_{i=1}^{I}\sum_{j=1}^{J}{\left(n_{ij}-\frac{1}{n}n_{i\cdot }n_{\cdot j}\right)}^{2}\\ & =\frac{1}{n^{2}}\sum_{i=1}^{I}\sum_{j=1}^{J}{\left(n_{ij}-n_{ij}^{*}\right)}^{2}, \end{array}$$

which is what we wanted to achieve.

Now, to start the way toward the asymptotic null distribution, let Z be either {1,…,I} or {1,…,J}. Then the discrete metric on Z

$$d\left(z,z^{\prime }\right)=1-\delta_{zz^{\prime }},$$

is dual to the following kernel in the sense of Sejdinovic et al. (2013):

$$k\left(z,z^{\prime }\right)=\delta_{zz^{\prime }},$$

which is known as the *discrete kernel*. Then clearly one can take the dummy function on each of X and Y as a feature map of the corresponding kernel/distance. We will denote them by $\phi :X⟶R^{I}$ and $\psi :Y⟶R^{J}$, where

$$\phi_{i}\left(X\right)=1_{\left\{X=i\right\}},\;\psi_{j}\left(Y\right)=1_{\left\{Y=j\right\}}.$$

Now we construct matrices $U={\left(U_{ij}\right)}_{n\times I}$ and $V={\left(V_{ij}\right)}_{n\times J}$ by transforming the X and Y samples with the feature maps:

$$U_{ki}=\phi_{i}\left(X_{k}\right)\;V_{kj}=\psi_{j}\left(Y_{k}\right).$$

Note that each of row of the previous matrices contains an observation of ϕ(X)∼Multi-Bernoulli(q) or ψ(Y)∼Multi-Bernoulli(r) (respectively). Therefore

$$1^{t}U∼{\mathrm{Multinomial}}_{I}\left(n,q\right)$$

$$1^{t}V∼{\mathrm{Multinomial}}_{J}\left(n,r\right).$$

Now, applying eq. (3) in Edelmann and Goeman (2022) to our feature maps, we get

$$n\;{\hat{\mathrm{dCov}}}_{\text{discrete}}^{2}\left(X,Y\right)=\frac{1}{n}\sum_{i=1}^{I}\sum_{j=1}^{J}{\left[U^{t}\left(I_{n}-H\right)V\right]}_{ij}^{2},$$

where $I_{n}$ is the n×n identity matrix and $H=\frac{1}{n}11^{t}$ has constant entries equal to $\frac{1}{n}$. If we now define $C\equiv {\left(C_{ij}\right)}_{I\times J}:=\frac{1}{\sqrt{n}}U^{t}\left(I_{n}-H\right)V$, we can compactly write our test statistic as a trace

$$n\;{\hat{\mathrm{dCov}}}_{\text{discrete}}^{2}\left(X,Y\right)=\mathrm{tr}\left[CC^{t}\right]=\mathrm{tr}\left[C^{t}C\right]=\sum_{i=1}^{I}\sum_{j=1}^{J}C_{ij}^{2}.$$

Expressing an empirical distance covariance as a trace of a matrix product, as we did above, is not unusual (Székely and Rizzo 2017) and indeed it is a very computationally efficient way of evaluating it. Nonetheless, for continuing the proof we are going to write:

$$n\;{\hat{\mathrm{dCov}}}_{\text{discrete}}^{2}\left(X,Y\right)=c^{t}c;$$

where $c:=\mathrm{vec}\left(C\right)\in R^{IJ}$ is the vectorization of matrix C (i.e., its image by the linear isomorphism $R^{I\times J}≅R^{IJ}$).

If one adds a vector with constant components a=a1 to a column or row of a matrix, the result of centering it with matrix I−H will be the same. Therefore, we can expand C as

$$C=\frac{1}{\sqrt{n}}\left(U^{t}-q1^{t}\right)\left(I-H\right)\left(V-1r^{t}\right)=$$

$$=\frac{1}{\sqrt{n}}\left(U^{t}-q1^{t}\right)\left(V-1r^{t}\right)-\frac{1}{n^{3/2}}\left(U^{t}-q1^{t}\right)11^{t}\left(V-1r^{t}\right).$$

The second term of the previous sum is

$$\begin{array}{ccc} D: & = & \frac{1}{\sqrt{n}}\;\left[\frac{1}{\sqrt{n}}\left(\begin{array}{c} \sum_{m=1}^{n}\left(\phi_{1}\left(X_{m}\right)-q_{1}\right)\\ \text{…}\\ \sum_{m=1}^{n}\left(\phi_{I}\left(X_{m}\right)-q_{I}\right) \end{array}\right)\right]\\ & & \times \;\left[\frac{1}{\sqrt{n}}\left(\sum_{m=1}^{n}\left(\psi_{1}\left(Y_{m}\right)-r_{1}\right),\text{…},\sum_{m=1}^{n}\left(\psi_{J}\left(Y_{m}\right)-q_{J}\right)\right)\right]. \end{array}$$

By the central limit theorem, it is easy to see that each entry $D_{ij}$ of D converges in probability to zero, owing to the fact that

$$\frac{1}{\sqrt{n}}\sum_{m=1}^{n}\left(\phi (X_{m})-q\right)\overset{D}{⟶}N_{I}\left(0,A\right)$$

$$\frac{1}{\sqrt{n}}\sum_{m=1}^{n}\left(\psi (Y_{m})-r\right)\overset{D}{⟶}N_{J}\left(0,B\right).$$

Hence, vec(D) converges in probability to the IJ‐dimensional null vector, and the limit in distribution of c will be that of the vectorization of

$$E:=\frac{1}{\sqrt{n}}\left(U^{t}-q1^{t}\right)\left(V-1r^{t}\right).$$

We can write the (i,j)th entry of the previous matrix as: $E_{ij}=\frac{1}{\sqrt{n}}\sum_{m=1}^{n}G_{mij}$, where

$$G_{mij}=\left(\phi_{i}\left(X_{m}\right)-q_{i}\right)\left(\psi_{j}\left(Y_{m}\right)-r_{j}\right).$$

Now, we see that we can apply the CLT to

$$\mathrm{vec}\left(E\right)=\frac{1}{\sqrt{n}}\sum_{m=1}^{n}\mathrm{vec}\left(G_{m}\right).$$

For fixed m∈{1,…,n}, let us see how the first and second moments of $\mathrm{vec}\left(G\right)\equiv \mathrm{vec}\left(G_{m}\right)$ look like. For i∈{1,…,IJ}, the ith component of E[vec(G)] vanishes under the null hypothesis (i.e., independence of X and Y):

$$\begin{array}{ccc} & & E[G_{\left(i-1\right)\%I+1,\left\lceil i/I\right\rceil }]\\ & & \;=E\left[\left(\phi_{(i-1)\%I+1}\left(X\right)-q_{(i-1)\%I+1}\right)\right]E\left[\left(\psi_{\left\lceil i/I\right\rceil }\left(Y\right)-r_{\left\lceil i/I\right\rceil }\right)\right]=0\cdot 0=0. \end{array}$$

We have used the notation % to indicate the remainder of an integer division, and $\left\lceil \cdot \right\rceil$ for the ceiling.

The (i,j)th entry of the variance–covariance matrix of vec(G) is

$$\begin{array}{ccc} & & \mathrm{Cov}(G_{\left(i-1\right)\%I+1,\left\lceil i/I\right\rceil },G_{\left(j-1\right)\%J+1,\left\lceil j/J\right\rceil })\\ & & \;=E[\left(\phi_{(i-1)\%I+1}\left(X\right)-q_{(i-1)\%I+1}\right)\left(\phi_{(j-1)\%J+1}\left(X\right)-q_{(j-1)\%J+1}\right)]\\ & & \;\times E[\left(\psi_{\left\lceil i/I\right\rceil }\left(Y\right)-r_{\left\lceil i/I\right\rceil }\right)\left(\psi_{\left\lceil j/J\right\rceil }\left(Y\right)-r_{\left\lceil j/J\right\rceil }\right)]\\ & & \;=a_{(i-1)\%I+1,(j-1)\%J+1}\;b_{\left\lceil i/I\right\rceil ,\left\lceil j/J\right\rceil }={\left[B⊗A\right]}_{ij}\;, \end{array}$$

with ⊗ denoting the Kronecker product.

Applying the central limit theorem once more, we get the limiting distribution of c:

$$c\overset{D}{⟶}N_{IJ}\left(0,\Gamma \right);\;\Gamma =B⊗A.$$

Now, one would be tempted to take Γ to the $-\frac{1}{2}$ and standardize c, but the reality is that Γ is never of full rank because A and B never are. So we are going to first take some sort of matrix root and then consider its inverse, instead of the other way round.

Let us write $\Gamma =MM^{t}$, where $M\in R^{IJ\times r}$ has rank r:=rank(Γ)≤IJ. If $M^{+}$ denotes the Moore–Penrose (pseudo)inverse of M, we can easily conclude that

$$w:=M^{+}c\overset{D}{⟶}N_{r}\left(0,I\right)$$

by taking into account that

$$M^{+}\Gamma {\left(M^{+}\right)}^{t}=M^{+}M{\left(M^{+}M\right)}^{t}=M^{+}MM^{+}M=M^{+}M=I_{r},$$

with the last equality owing to the fact of M having full column rank.

We can finally go back to the expression of the empirical distance covariance:

$$n\;{\hat{\mathrm{dCov}}}_{\text{discrete}}^{2}\left(X,Y\right)=w^{t}\Gamma w.$$

As Γ is symmetric, we can diagonalize it with an orthogonal modal matrix $Q\in R^{IJ\times IJ}$:

$$\Gamma =Q^{t}\Lambda Q,$$

where $\Lambda \in R^{IJ\times IJ}$ is a diagonal matrix and has the eigenvalues of B⊗A in its diagonal (which are the IJ products of the eigenvalues ${\left\{\lambda_{i}\right\}}_{i}$ and ${\left\{\mu_{j}\right\}}_{j}$ of A and B, respectively). This allows us to conclude

$$n\;{\hat{\mathrm{dCov}}}_{\text{discrete}}^{2}\left(X,Y\right)\overset{D}{⟶}\sum_{i,j}\lambda_{i}\mu_{j}Z_{ij}^{2},$$

where ${\left\{Z_{ij}\right\}}_{i,j}$ are IID standard Gaussian.□

## Appendix B Proof of Theorem 3.1

We will first derive the compact expression of $E_{n}$. To that purpose, we first recall the definition of energy distance:

(B1)
$$E_{n}=n\left[\frac{2}{n}\sum_{l=1}^{n}Ed\left(x_{l},X\right)-Ed\left(X,X^{\prime }\right)-\frac{1}{n^{2}}\sum_{l,m=1}^{n}d\left(x_{l},x_{m}\right)\right];$$

where all the notation so far is the same as in the main manuscript.

We first note that, for the discrete metric: $Ed\left(x_{l},X\right)=P\left\{X\neq x_{l}\right\}$. Summing over l and multiplying by $\frac{2}{n}$:

$$\frac{2}{n}\sum_{l=1}^{n}Ed\left(x_{l},X\right)=\frac{2}{n}\sum_{l=1}^{n}\left(1-P\left\{X=x_{l}\right\}\right)=\sum_{i=1}^{I}\frac{n_{i}}{n}\left(1-p_{i}\right)=\sum_{i=1}^{I}{\hat{p}}_{i}\left(1-p_{i}\right);$$

where ${\hat{p}}_{i}:=\frac{n_{i}}{n}$ is the estimated probability of category i∈{1,…,I} given the sample.

Second, we write the straightforward identity

$$Ed\left(X,X^{\prime }\right)=1-\sum_{i=1}^{I}p_{i}^{2}.$$

And finally, for the remaining term of $E_{n}/n$, we apply similar arguments to conclude:

$$\frac{1}{n^{2}}\sum_{l,m=1}^{n}d\left(x_{l},x_{m}\right)=1-\sum_{i=1}^{I}{\hat{p}}_{i}^{2}.$$

Now, adding up the three expressions:

$$\frac{E_{n}}{n}=2\sum_{i=1}^{I}{\hat{p}}_{i}\left(1-p_{i}\right)-\left[1-\sum_{i=1}^{I}p_{i}^{2}\right]-\left[1-\sum_{i=1}^{I}{\hat{p}}_{i}^{2}\right]=$$

$$-2\sum_{i=1}^{I}{\hat{p}}_{i}p_{i}+\sum_{i=1}^{I}p_{i}^{2}+\sum_{i=1}^{I}{\hat{p}}_{i}^{2}=\sum_{i=1}^{I}{\left({\hat{p}}_{i}-p_{i}\right)}^{2}=\frac{1}{n^{2}}\sum_{i=1}^{I}{\left(n_{i}-n_{i}^{*}\right)}^{2}.$$

We will now derive the asymptotic null distribution of V‐statistic $E_{n}$ from classical U‐statistic theory (our V‐statistic is a U‐statistic plus an asymptotically constant term). By conveniently working out expression B1, we get

$$\begin{array}{ccc} E_{n}/n & = & \frac{1}{n^{2}}\sum_{l,m=1}^{n}\left[-d\left(x_{l},x_{m}\right)+Ed\left(x_{l},X\right)+Ed\left(x_{m},X\right)-Ed\left(X,X^{\prime }\right)\right]\\ & \equiv & \frac{1}{n^{2}}\sum_{l,m=1}^{n}h\left(x_{l},x_{m}\right); \end{array}$$

where we define h as the symmetric function:

$$h\left(y,z\right):=-d\left(y,z\right)+Ed\left(y,X\right)+Ed\left(z,X\right)-Ed\left(X,X^{\prime }\right).$$

By grouping the terms:

$$E_{n}/n=\frac{1}{n^{2}}\sum_{l\neq m}h\left(x_{l},x_{m}\right)+\frac{1}{n^{2}}\sum_{l=1}^{n}Ed\left(x_{l},X\right)-\frac{1}{n}Ed\left(X,X^{\prime }\right).$$

Now multiplying both sides by n, the following expression for the energy distance arises:

(B2)
$$E_{n}=\frac{n(n-1)}{n^{2}}\;n\;U+\frac{1}{n}\sum_{i=1}^{I}{\hat{p}}_{i}\left(1-p_{i}\right)-Ed\left(X,X^{\prime }\right).$$

Applying the unnumbered theorem in Section 5.5.2 of Serfling (1980), we see that

$$n\;U\overset{D}{⟶}\sum_{i=1}^{I}\lambda_{i}\left(Z_{i}^{2}-1\right)$$

as n→∞, where we note that $U=\frac{1}{n(n-1)}\sum_{l\neq m}h\left(x_{l},x_{m}\right)$ is a U‐statistic and ${\left\{\lambda_{i}\right\}}_{i}$ is the spectrum of matrix

$$C={\left(p_{i}\delta_{ij}-p_{i}p_{j}\right)}_{I\times I}.$$

Summing the elements of its diagonal yields its trace

$$\mathrm{tr}\left(C\right)=\sum_{i=1}^{I}\left(p_{i}-p_{i}^{2}\right)=1-\sum_{i=1}^{I}p_{i}^{2}=Ed\left(X,X^{\prime }\right).$$

We finally see that the middle term in (B2) converges in distribution to 0 under the null, owing to the fact that ${\hat{p}}_{i}\overset{a.s.}{\underset{n\to \infty }{⟶}}p_{i}$ by the strong law of large numbers. In conclusion:

$$E_{n}\overset{D}{⟶}\sum_{i=1}^{I}\lambda_{i}\left(Z_{i}^{2}-1\right)+\sum_{i=1}^{I}\lambda_{i}=\sum_{i=1}^{I}\lambda_{i}Z_{i}^{2},$$

where ${\left\{Z_{i}^{2}\right\}}_{i=1}^{I}$ are IID chi‐squared variables with one degree of freedom each.□
