# Supplementary material for: Tests for Categorical Data Beyond Pearson: A Distance Covariance and Energy Distance Approach
Source: Biom J. 2026 Jun 8;68(3):e70129. doi: 10.1002/bimj.70129 (PMC13244122; doi:10.1002/bimj.70129)
Supplement: Supplementary file 1 — Supporting File: bimj70129‐sup‐0001‐DataCode.zip. [file BIMJ-68-e70129-s001.zip › README.docx]

**Title:** Tests for categorical data beyond Pearson: A distance covariance and energy distance approach

**Authors:** Fernando Castro-Prado, Wenceslao Gonzalez-Manteiga, Javier Costas,

Fernando Facal and Dominic Edelmann

# Corresponding Author for Code:

Name: Fernando Castro-Prado

Email: [f](mailto:raynerrui@ruc.edu.cn).castro.prado@pm.me

# Configurations:

# After running the session info command, we get the following information on our R session.

> sessionInfo()

R version 4.3.1 (2023-06-16 ucrt)

Platform: x86_64-w64-mingw32/x64 (64-bit)

Running under: Windows 10 x64 (build 19045)

Matrix products: default

locale:

[1] LC_COLLATE=Spanish_Spain.utf8 LC_CTYPE=Spanish_Spain.utf8

[3] LC_MONETARY=Spanish_Spain.utf8 LC_NUMERIC=C

[5] LC_TIME=Spanish_Spain.utf8

time zone: Europe/Madrid

tzcode source: internal

attached base packages:

[1] stats graphics grDevices utils datasets methods base

other attached packages:

[1] ggplot2_3.4.3 momentchi2_0.1.5 coga_1.2.0

loaded via a namespace (and not attached):

[1] utf8_1.2.3 R6_2.5.1 magrittr_2.0.3 gtable_0.3.4 glue_1.6.2

[6] tibble_3.2.1 pkgconfig_2.0.3 lifecycle_1.0.3 cli_3.6.1 fansi_1.0.5

[11] scales_1.2.1 grid_4.3.1 vctrs_0.6.3 withr_2.5.1 compiler_4.3.1

[16] tools_4.3.1 pillar_1.9.0 munsell_0.5.0 Rcpp_1.0.11 colorspace_2.1-0

[21] rlang_1.1.1

# General information

# Our paper presents testing procedures for two separate problems with categorical data — independence of two variables, and goodness of fit of one variable to a given distribution. There are, therefore, numerical examples for each of those tests, both in simulated and real datasets.

# The rest of the present README file explains how to reproduce the simulations (calibration of the type I error and power curves, comparing with competing methods) and the real data analyses (chronicity of schizophrenia and Hardy–Weinberg equilibrium) in our manuscript. For each of these tasks, one will find the necessary files in a subfolder with a straightforward name within the *Code_and_Data* folder.

# Reproducing the simulations

# For the simulations, the reader should refer to subfolder */simu/* . In it, there is an R script called *test_functions_ct_dcov.R* that contains most of the necessary testing functions for both problems (i.e., independence and goodness of fit). An exception is the competing method USP, which also depends on functions defined in *test_functions_uspa.R*.

# For independence testing, the simulations are generated by running *simu_indep_with_plots.R*, which

# also provides plots for the power curve comparison of our methodology with competitors (producing *Figure 2.pdf* as its output). All its numerical results are stored in plain text files within the */pvals/* subfolder. *Figure 1* of the manuscript can be generated as PDF by running the script called *plotting_calibration_methods_indep.R*.

# When it comes to testing for goodness of fit, the numerical results are crunched in *simu_gof.R*. Power plots (*Figures 4* and *6*) are created by sourcing *plotting_power_gof.R*. On the other hand, *Figures 3* and *5*, that illustrate type I error control can be generated with *plotting_calibration_gof.R*.

# Reproducing the real data analyses

# As with the simulations, here we do everything twice, once for independence and then again for goodness of fit. We will be referring to subfolder *real_data* of the main *Code_and_Data* folder.

# For independence, we provide the dataset for the example on admission history of schizophrenia

# patients in *admission_data.txt*. It can be then analysed by running *admission.R*. Relevant results and

# intermediate steps are marked as comments in that script. *Table 1.txt* is produced as an output and, as it was the case with figures, its numbering matches the one in the paper.

# For goodness of fit, the data of the allele frequencies is typed out inside the corresponding R

# scripts, and the external data file *pgc3_snps.txt* contains a list of SNPs known to be associated

# with schizophrenia, against which we check the variants that we consider in each example. The

# testing for Hardy–Weinberg equilibrium in a biallelic locus is carried out in *hwe_2allele.R*, whereas the triallelic setting is dealt with in *hwe_3allele.R*. The former script exports the table of expected cell counts under the null hypothesis for the biallelic locus and the latter, the table of observed cell counts for the triallelic case. Those two tables match the two unnumbered tables in our manuscript, both contained in Section 5.2.

# Source of data

The discrete variables we analyse are derived from Facal et al. (2021), as explained in the main manuscript. Since we are not showing identifiable individual-level patient data, there are no issues regarding data privacy when sharing our observations of processed discrete variables.
