# Supplementary figures and images for: Tests for Categorical Data Beyond Pearson: A Distance Covariance and Energy Distance Approach
Source: Biom J. 2026 Jun 8;68(3):e70129. doi: 10.1002/bimj.70129 (PMC13244122; doi:10.1002/bimj.70129)

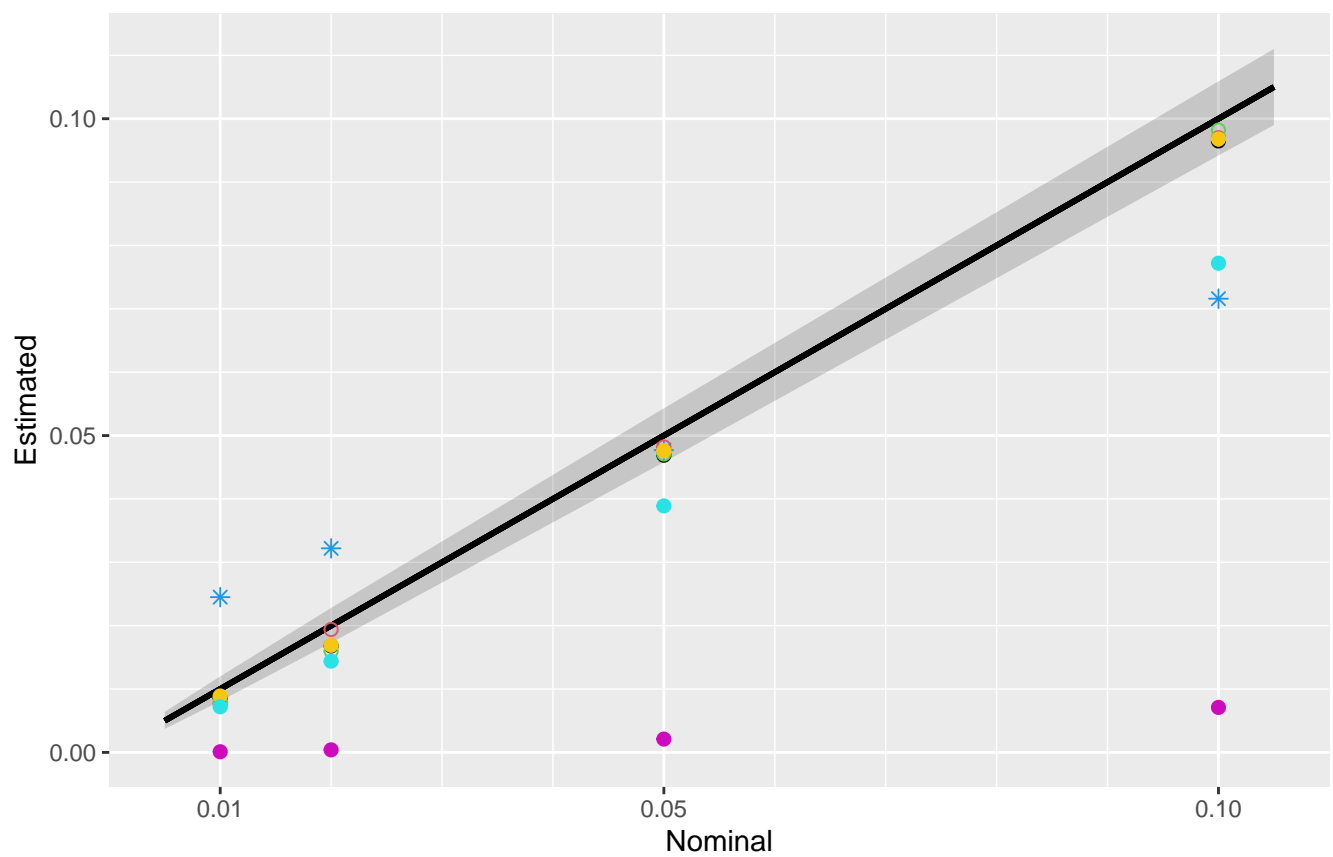

Supplement: Supplementary file 1 — Supporting File: bimj70129‐sup‐0001‐DataCode.zip. [file BIMJ-68-e70129-s001.zip › simu/Figure 1.pdf]

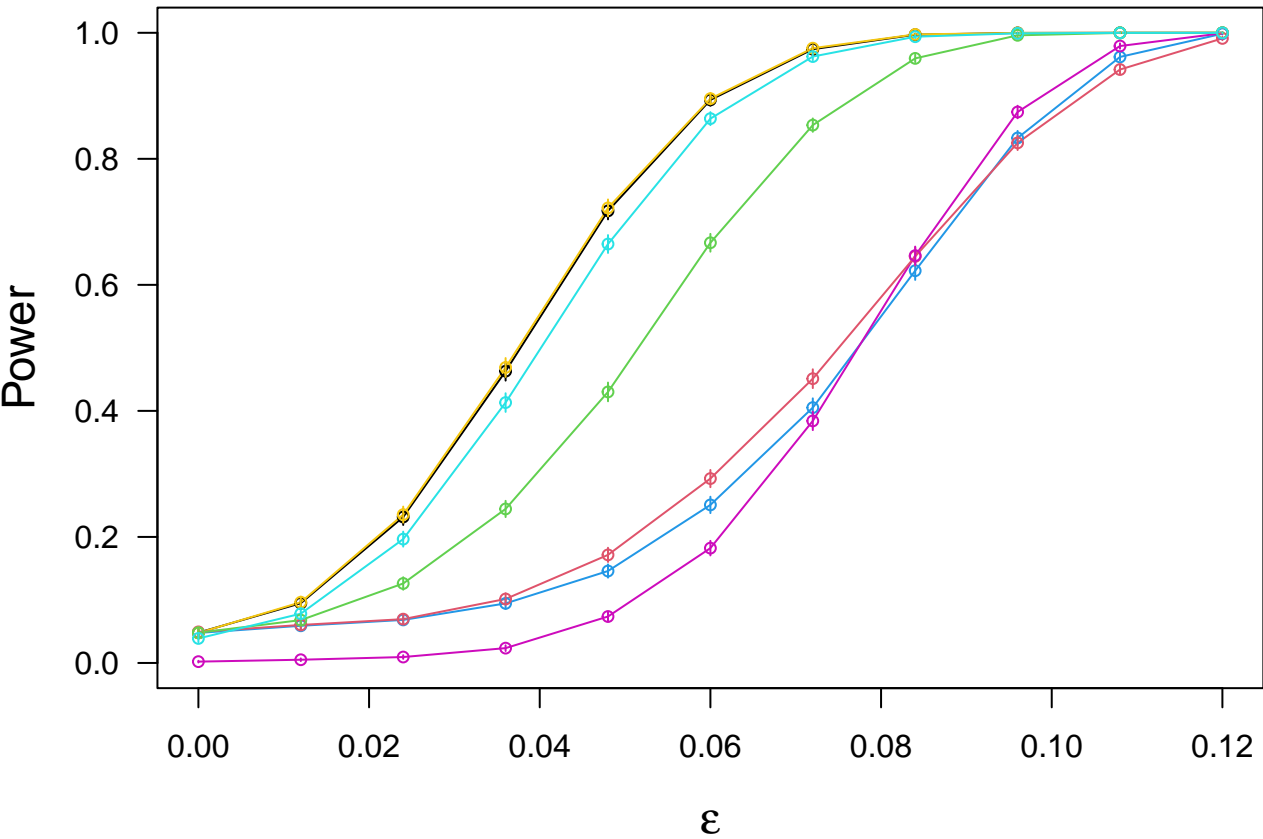

Supplement: Supplementary file 1 — Supporting File: bimj70129‐sup‐0001‐DataCode.zip. [file BIMJ-68-e70129-s001.zip › simu/Figure 2.pdf]

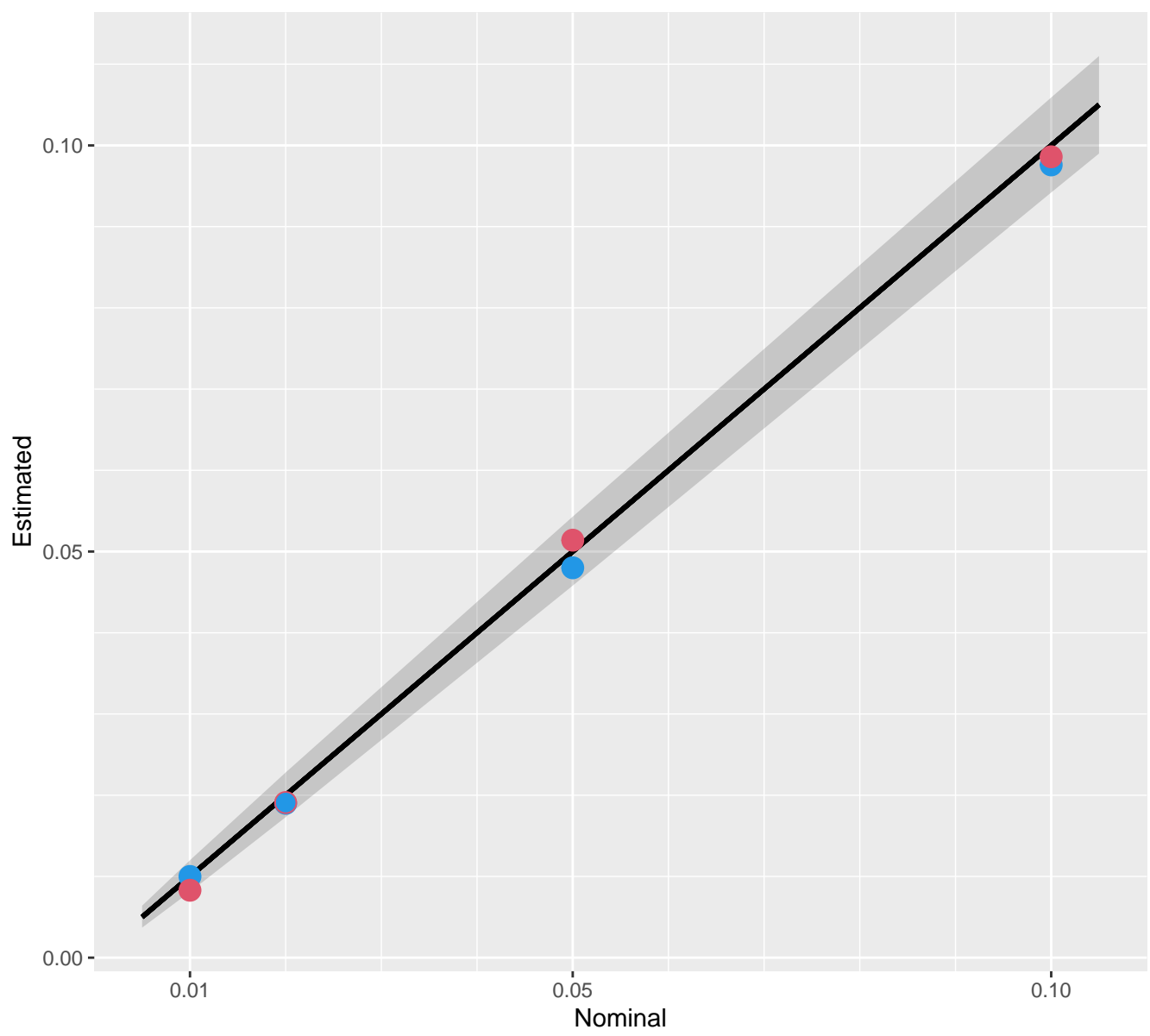

Supplement: Supplementary file 1 — Supporting File: bimj70129‐sup‐0001‐DataCode.zip. [file BIMJ-68-e70129-s001.zip › simu/Figure 3 a.pdf]

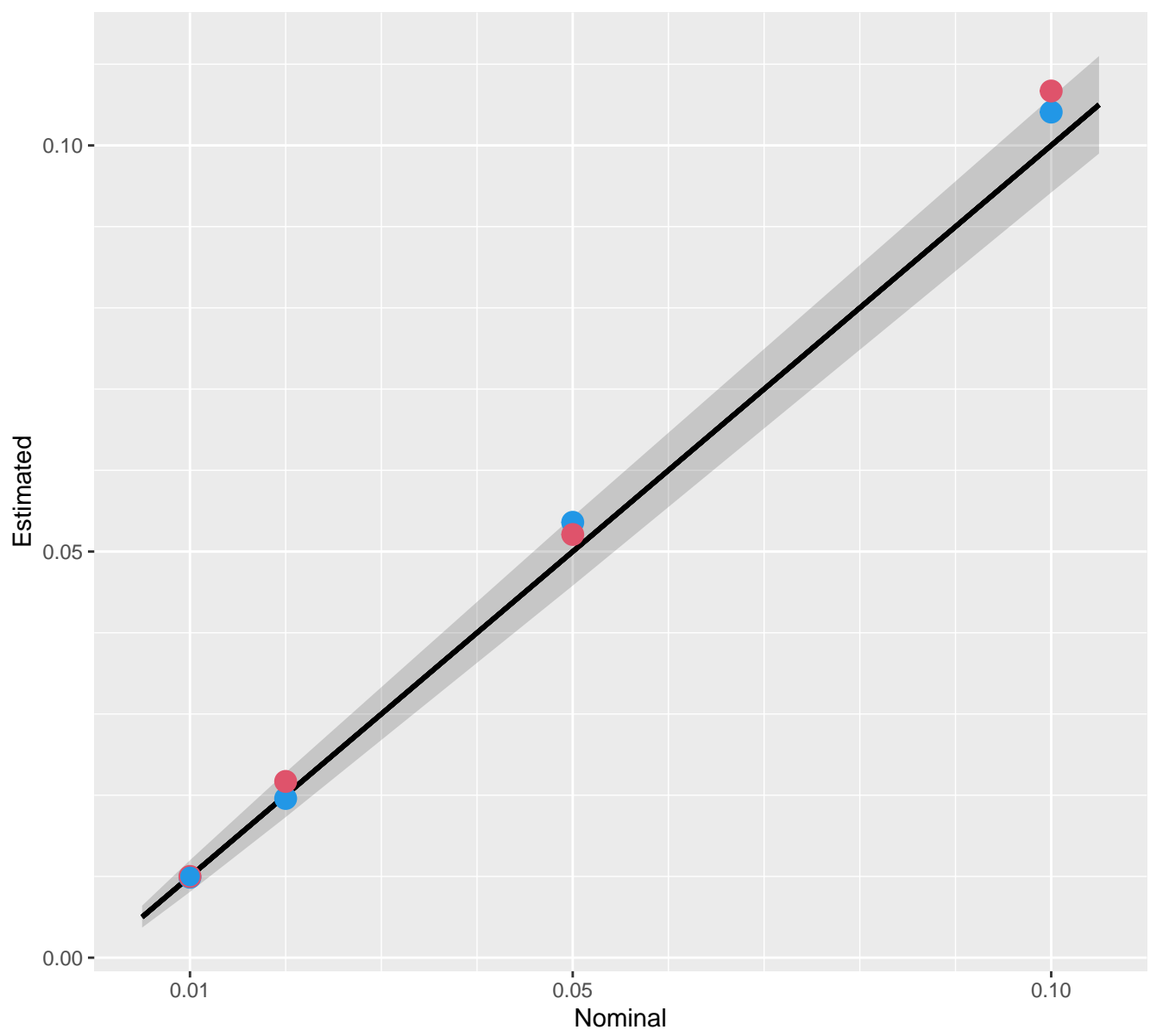

Supplement: Supplementary file 1 — Supporting File: bimj70129‐sup‐0001‐DataCode.zip. [file BIMJ-68-e70129-s001.zip › simu/Figure 3 b.pdf]

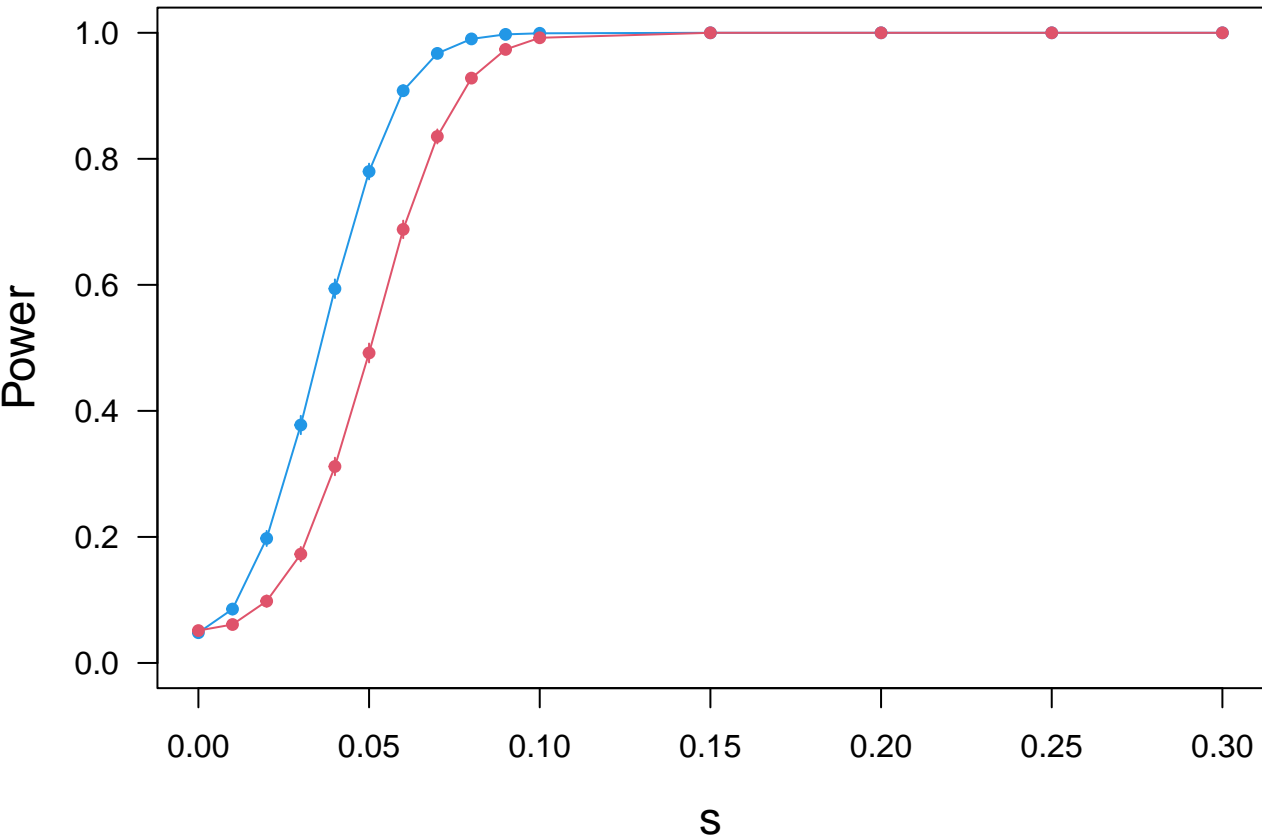

Supplement: Supplementary file 1 — Supporting File: bimj70129‐sup‐0001‐DataCode.zip. [file BIMJ-68-e70129-s001.zip › simu/Figure 4 a.pdf]

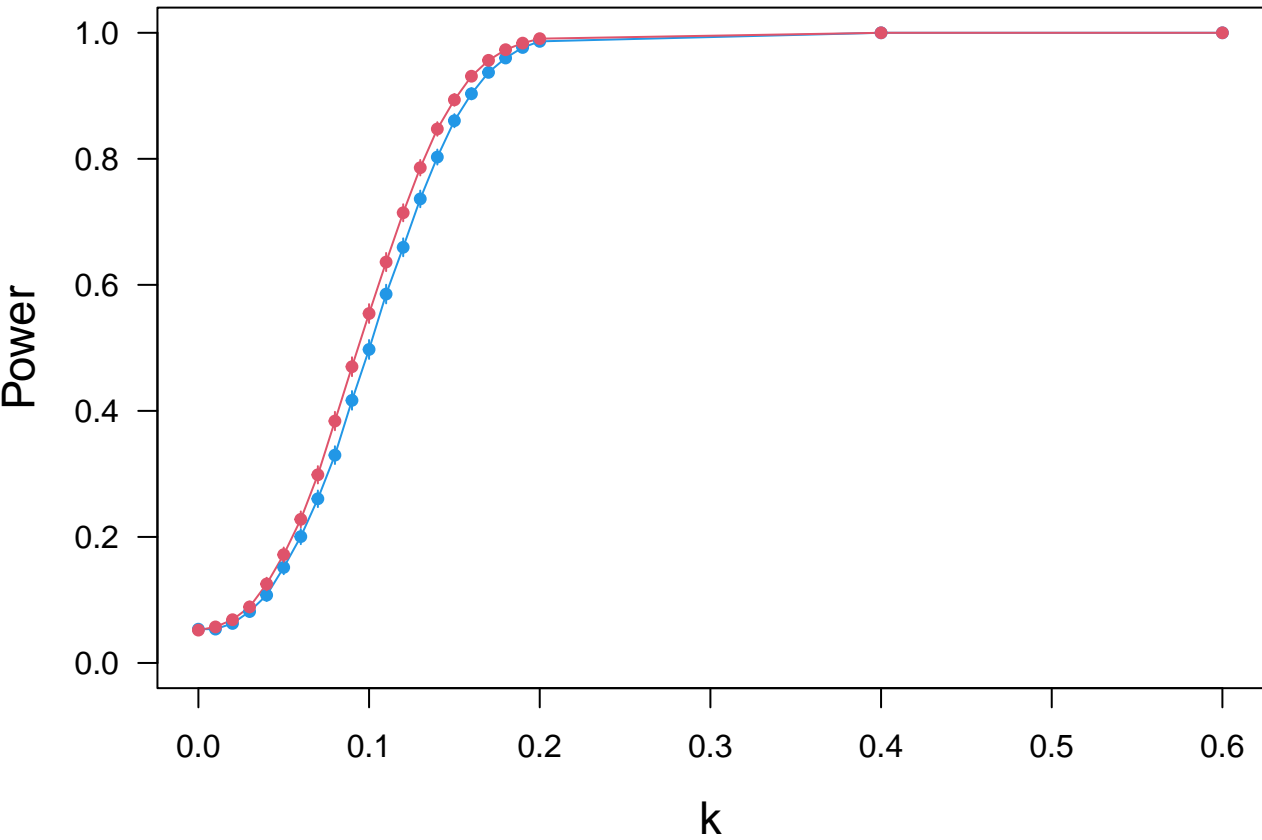

Supplement: Supplementary file 1 — Supporting File: bimj70129‐sup‐0001‐DataCode.zip. [file BIMJ-68-e70129-s001.zip › simu/Figure 4 b.pdf]

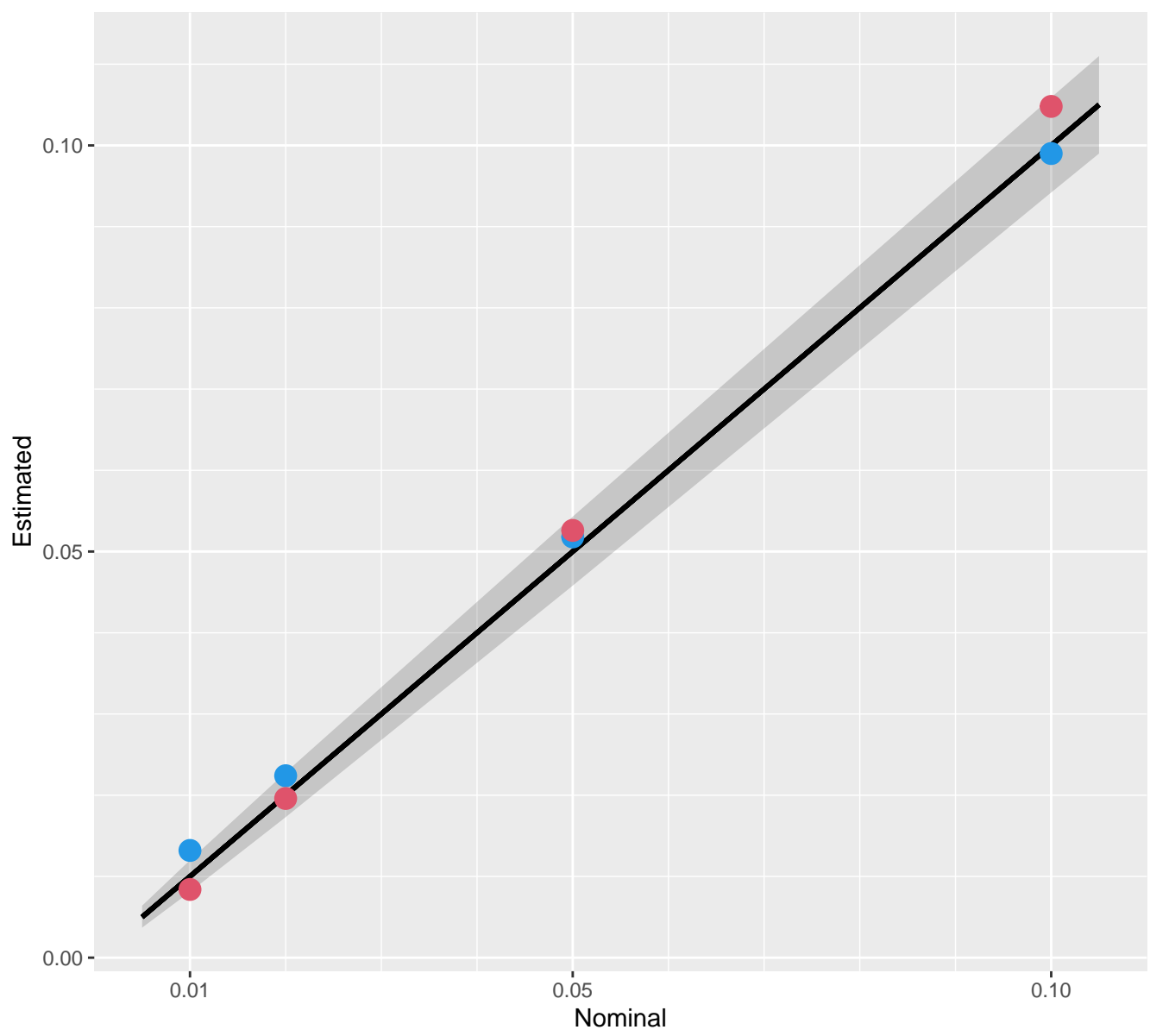

Supplement: Supplementary file 1 — Supporting File: bimj70129‐sup‐0001‐DataCode.zip. [file BIMJ-68-e70129-s001.zip › simu/Figure 5 a.pdf]

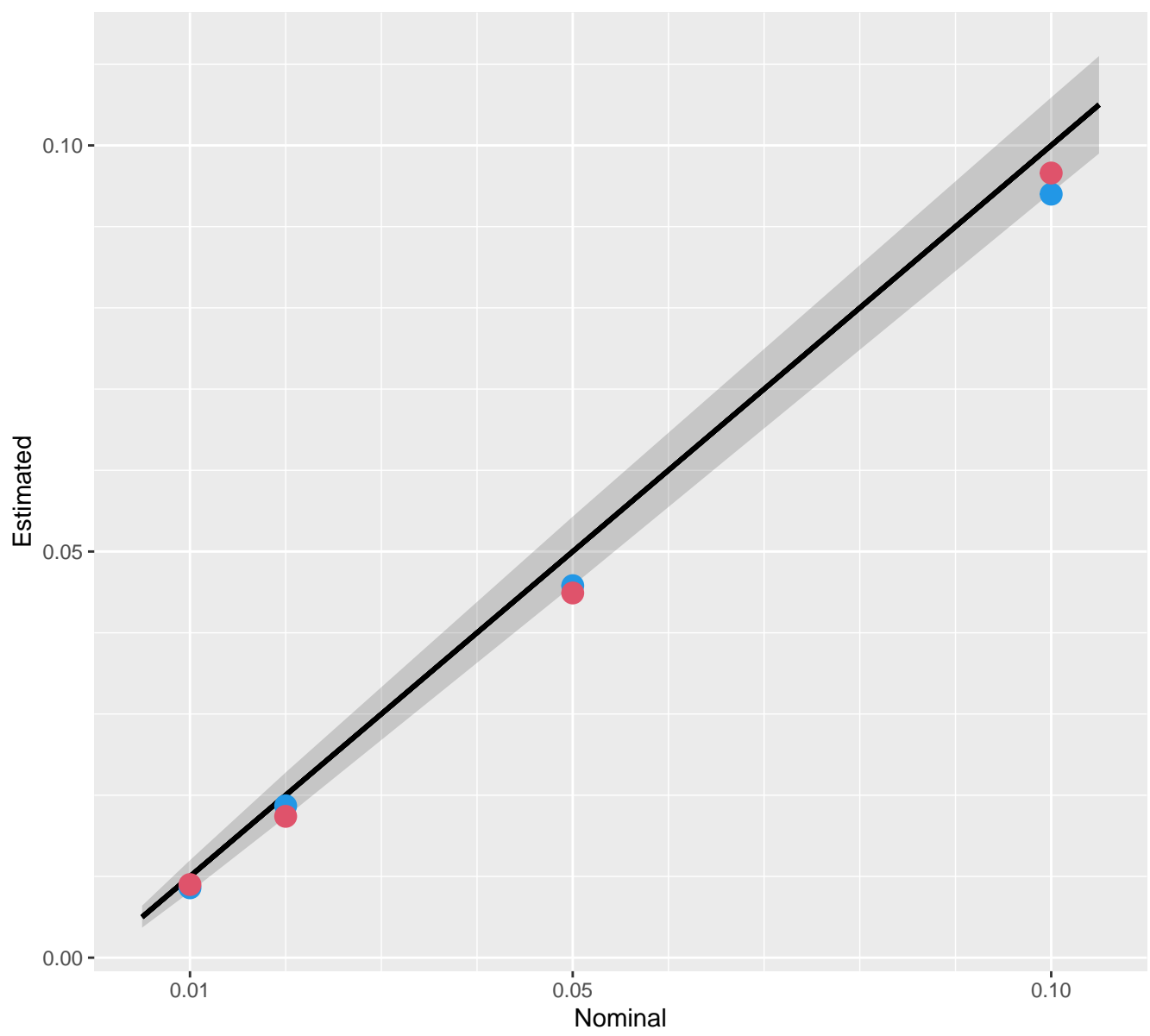

Supplement: Supplementary file 1 — Supporting File: bimj70129‐sup‐0001‐DataCode.zip. [file BIMJ-68-e70129-s001.zip › simu/Figure 5 b.pdf]

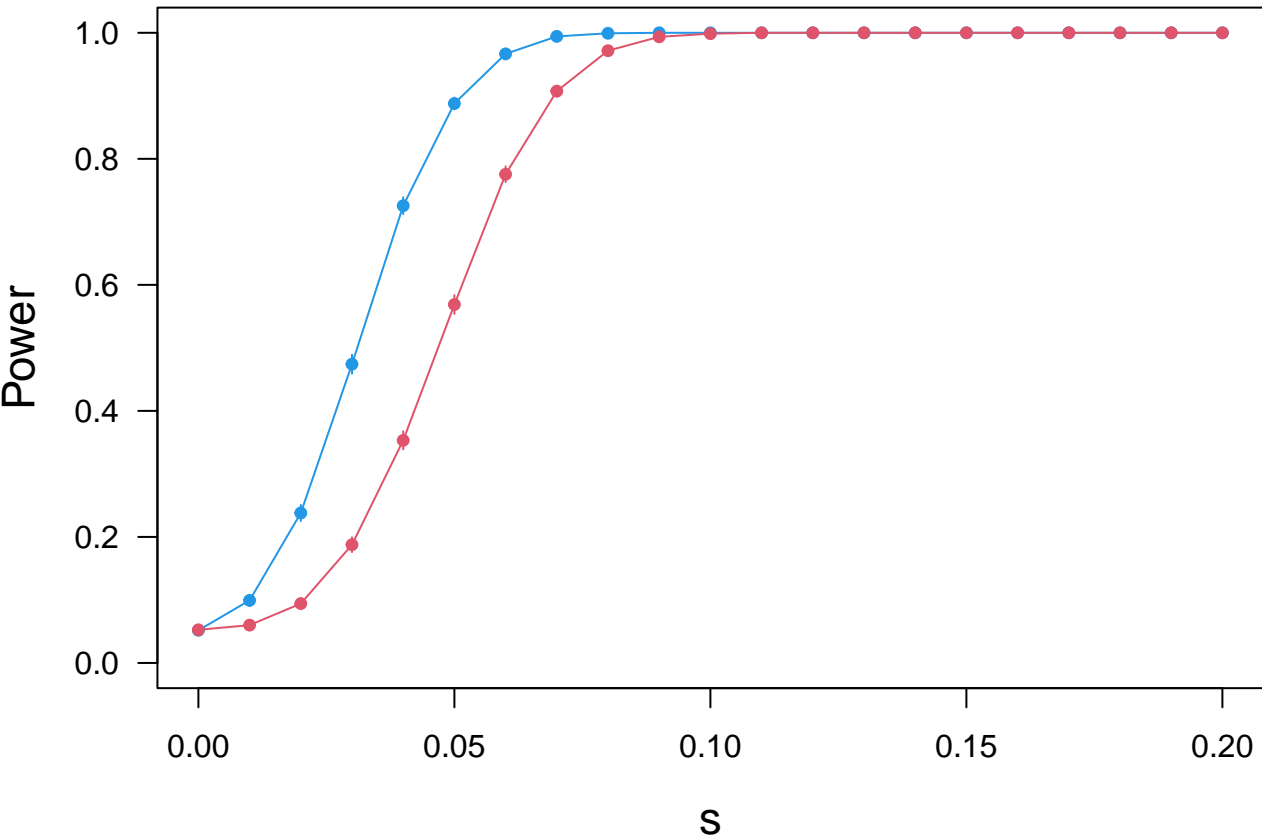

Supplement: Supplementary file 1 — Supporting File: bimj70129‐sup‐0001‐DataCode.zip. [file BIMJ-68-e70129-s001.zip › simu/Figure 6 a.pdf]

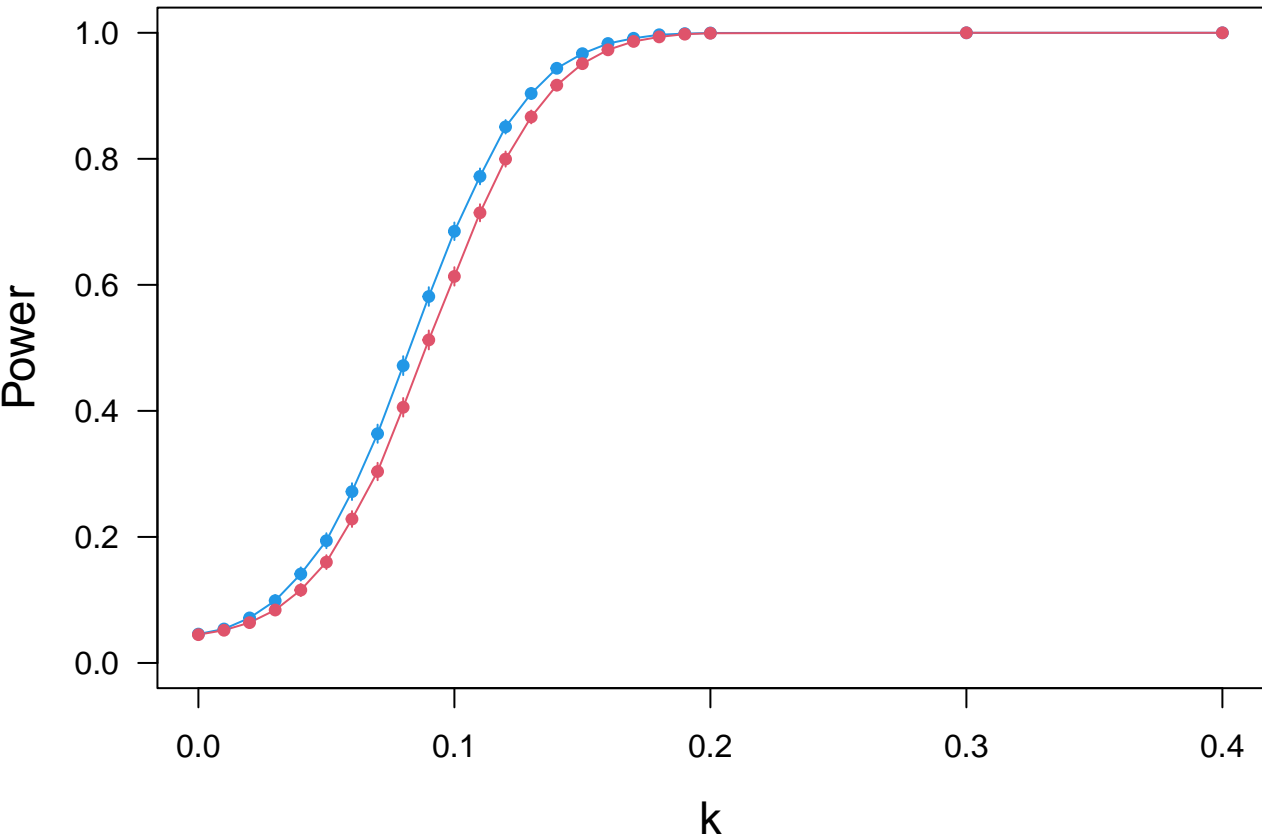

Supplement: Supplementary file 1 — Supporting File: bimj70129‐sup‐0001‐DataCode.zip. [file BIMJ-68-e70129-s001.zip › simu/Figure 6 b.pdf]
